# Susceptibility to *Vibrio cholerae* Infection in a Cohort of Household Contacts of Patients with Cholera in Bangladesh

**DOI:** 10.1371/journal.pntd.0000221

**Published:** 2008-04-09

**Authors:** Jason B. Harris, Regina C. LaRocque, Fahima Chowdhury, Ashraful I. Khan, Tanya Logvinenko, Abu S. G. Faruque, Edward T. Ryan, Firdausi Qadri, Stephen B. Calderwood

**Affiliations:** 1 Division of Infectious Diseases, Massachusetts General Hospital, Boston, Massachusetts, United States of America; 2 Department of Pediatrics, Massachusetts General Hospital, Boston, Massachusetts, United States of America; 3 Department of Medicine, Harvard Medical School, Boston, Massachusetts, United States of America; 4 International Centre for Diarrhoeal Disease Research, Dhaka, Bangladesh; 5 Department of Biostatistics, Massachusetts General Hospital, Boston, Massachusetts, United States of America; 6 Department of Immunology and Infectious Diseases, Harvard School of Public Health, Boston, Massachusetts, United States of America; 7 Department of Microbiology and Molecular Genetics, Harvard Medical School, Boston, Massachusetts, United States of America; Weill Medical College of Cornell University, United States of America

## Abstract

**Background:**

Despite recent progress in understanding the molecular basis of *Vibrio cholerae* pathogenesis, there is relatively little knowledge of the factors that determine the variability in human susceptibility to *V. cholerae* infection.

**Methods and Findings:**

We performed an observational study of a cohort of household contacts of cholera patients in Bangladesh, and compared the baseline characteristics of household members who went on to develop culture-positive *V. cholerae* infection with individuals who did not develop infection. Although the vibriocidal antibody is the only previously described immunologic marker associated with protection from *V. cholerae* infection, we found that levels of serum IgA specific to three V. *cholerae* antigens—the B subunit of cholera toxin, lipopolysaccharide, and TcpA, the major component of the toxin–co-regulated pilus—also predicted protection in household contacts of patients infected with *V. cholerae* O1, the current predominant cause of cholera. Circulating IgA antibodies to TcpA were also associated with protection from *V. cholerae* O139 infection. In contrast, there was no association between serum IgG antibodies specific to these three antigens and protection from infection with either serogroup. We also found evidence that host genetic characteristics and serum retinol levels modify susceptibility to *V. cholerae* infection.

**Conclusions:**

Our observation that levels of serum IgA (but not serum IgG) directed at certain *V. cholerae* antigens are associated with protection from infection underscores the need to better understand anti–*V. cholerae* immunity at the mucosal surface. Furthermore, our data suggest that susceptibility to *V. cholerae* infection is determined by a combination of immunologic, nutritional, and genetic characteristics; additional factors that influence susceptibility to cholera remain unidentified.

## Author Summary

*Vibrio cholerae* is the bacterium that causes cholera, a severe form of diarrhea that leads to rapid and potentially fatal dehydration when the infection is not treated promptly. Cholera remains an important cause of diarrhea globally, and *V. cholerae* continues to cause major epidemics in the most vulnerable populations. Although there have been recent discoveries about how the bacterium adapts to the human intestine and causes diarrhea, there is little understanding of why some people are protected from infection with *V. cholerae*. This article describes several factors that are associated with the risk of developing *V. cholerae* infection among people living in the same household with a patient with severe cholera who are at high risk of contracting the infection. One of the findings is that IgA antibodies, a type of antibody associated with immunity at mucosal surfaces such as the intestine, that target several components of the bacteria are associated with immunity to *V. cholerae* infection. This article also describes genetic and nutritional factors that additionally influence susceptibility to *V. cholerae* infection.

## Introduction

*Vibrio cholerae* causes a spectrum of infection in humans ranging from asymptomatic colonization to severe secretory diarrhea. *V. cholerae* is differentiated serologically by the O antigen of its lipopolysaccharide (LPS); the vast majority of human cholera is caused by the O1 and O139 serogroups. The O1 serogroup of *V. cholerae* is classified into two biotypes (classical and El Tor), and two major serotypes (Inaba and Ogawa) [1]. In the 1960s, the *V. cholerae* O1 El Tor biotype emerged as a major cause of cholera, ultimately replacing the classical biotype. In 1992, the *V. cholerae* O139 serogroup first appeared, and after briefly predominating in South Asia, now persists in this region, but at much lower levels than *V. cholerae* O1 El Tor.

Although absent from the view of most resource-rich nations, the global impact of *V. cholerae* infection remains enormous. Cholera is vastly underreported, but it is estimated that there are over one million cases of cholera annually, with more than 100,000 deaths [2]. According to the WHO, there is an urgent need for cholera vaccines that provide durable protection, particularly among children less than 5 years of age in developing countries [2]. One limitation to the development of effective vaccination programs for cholera is that the factors that determine human susceptibility to *V. cholerae* remain poorly defined.

Natural infection with *V. cholerae* O1 induces adaptive immune responses that are protective against subsequent disease. Volunteer studies in non-endemic settings have demonstrated that infection with classical biotype *V. cholerae* O1 provides 100% protection from subsequent challenge with a classical biotype strain, while infection with the El Tor biotype of *V. cholerae* O1 provides 90% protection from subsequent challenge with an El Tor strain. This protection lasts at least 3 years in volunteer studies [3]. In an endemic area, an initial episode of El Tor cholera reduces the risk of a second clinically apparent infection by 90% over the next several years [4].

The best-studied correlate of immunity to *V. cholerae* is the serum vibriocidal antibody, a complement-fixing bacteriocidal antibody. Seroepidemiologic studies in endemic areas have shown that vibriocidal antibody titers increase with age and correlate with protection from cholera [5],[6]. However, there is no threshold vibriocidal antibody titer above which complete protection from infection is achieved, and the vibriocidal antibody may be a surrogate marker for an undetermined protective immune response at the mucosal surface [7].

Although a major component of the vibriocidal antibody is directed against serotype specific LPS, levels of serum LPS-specific immunoglobulin G (IgG) antibody have not been found to correlate with protection from cholera in humans [5]. A portion of the vibriciodal antibody may also be directed against *V. cholerae* outer membrane proteins [8]. Antitoxin immunity is primarily directed at the B subunit of cholera toxin (CTB); however serum IgG-antibodies to CTB have not been found to correlate with protection from cholera [5], and toxin-based vaccines confer only transient protection [9]. Robust mucosal and systemic humoral responses to TcpA, the major subunit of the toxin-coregulated pilus (TCP), a type IV pilus that is required for intestinal colonization, have recently been demonstrated in patients with cholera [10], but it is unknown whether these responses are associated with protection from disease.

In addition to adaptive immune responses, innate host characteristics may also influence the outcome of an individual's exposure to *V. cholerae*. Multiple case-control studies in cholera endemic areas have demonstrated that individuals with blood group O are at increased risk of hospitalization with cholera, and it has been hypothesized that *V. cholerae* infection may have selected for the low prevalence of the O blood group in the Ganges Delta region, a historic and current global epicenter of cholera [11]. Other genetic or innate immune factors may also influence susceptibility to cholera. For example, studies of duodenal biopsies obtained from patients with cholera demonstrate recruitment of neutrophils to the intestinal epithelium during acute infection [12], and also reveal increased expression of broad classes of innate immune effectors, including lactoferrin and other antibacterial proteins [13], suggesting further potential sources of genetic variability that might contribute to susceptibility to cholera.

The nutritional status of the host may also affect susceptibility to diarrheal diseases such as cholera, by influencing both innate and adaptive immunity. The micronutrients zinc and vitamin A play central roles in mucosal immunity, and when given in community-based supplementation programs, may reduce the incidence and morbidity of diarrheal diseases [14]. Although zinc supplementation in children has been found to modify adaptive immune responses to *V. cholerae* [15],[16], there are no prior studies demonstrating a specific association between baseline nutritional characteristics and the risk of *V. cholerae* infection in individuals.

Here, we present the results of a prospective, observational study aimed at identifying host factors that are associated with *V. cholerae* infection within households in a cholera-endemic setting. Our results may be relevant to the design and evaluation of more effective cholera vaccines for use in resource-poor areas of the world.

## Methods

### Study design and subject enrollment

The hospital at the Clinical Research and Service Centre (CRSC) of the International Centre for Diarrhoeal Disease Research, Bangladesh (ICDDR,B) provides care for more than 100,000 patients annually, including approximately 10,000 to 20,000 with cholera, the majority of whom are residents of Dhaka city. Index cases presenting to the hospital with severe acute watery diarrhea were eligible for inclusion in this study if their stool cultures were subsequently positive for *V. cholerae*, if they were older than 6 months, and if they were without significant co-morbid conditions. Upon presentation of the index case, a field team discussed enrollment with household contacts of the index case, and consenting household contacts were then enrolled into the study immediately upon culture confirmation of the index case (within 24 hours of presentation of the index case). Household contacts were defined as individuals who shared the same cooking pot for three or more days. Blood specimens for ABO typing, baseline vibriocidal titers, and baseline anti-LPS, anti-CTB and anti-TcpA antibody levels were immediately collected from consenting household contacts upon enrollment. The field team visited household contacts on each of the next six days, and again on days 14 and 21. During these visits, contacts were questioned about diarrheal symptoms, and rectal swabs were obtained for *V. cholerae* culture. Follow-up blood samples for vibriocidal antibody titers were obtained from contacts on study days 7 and 21. All patients designated as having completed follow-up had serum successfully obtained at baseline and day 21, and 98% of the interim field visits resulted in collection of clinical data and rectal swabs.

Household contacts were excluded from the analysis if they did not complete 21 days of follow-up. Household contacts were also independently excluded from the analysis of their baseline immunologic characteristics if they had symptoms of diarrhea during the week preceding enrollment, if they had a positive rectal swab culture for *V. cholerae* on enrollment into the study, or if they developed infection with *V. cholerae* of a serogroup or serotype that did not correspond to that of the index case.

Informed consent for participation in this research was obtained from participants or their guardians. The human experimentation guidelines of the U.S. Department of Health and Human Services were followed in the conduct of this research. Approval for this human study was obtained from the Institutional Review Board of the Massachusetts General Hospital and the Research and the Ethical Review Committees of the ICDDR,B.

### Confirmation of bacterial strains

All index cases of cholera were confirmed by culturing stool for *V. cholerae* on taurocholate-tellurite-gelatin agar (TTGA). After overnight incubation of plates, serological confirmation of suspected *V. cholerae* colonies was carried out by slide agglutination [17],[18]. Rectal swab specimens from household contacts were collected in Cary-Blair transport media for subsequent plating on TTGA and colony identification as above. *V. cholerae* was the only pathogen for which microbiologic screening was carried out during the 21 day follow-up period.

### Immunologic and other laboratory assays

Vibriocidal antibody assays were performed with methodology previously described, using guinea pig complement and the homologous serogroup/type of *V. cholerae* O1 El Tor Ogawa (strain 25049), *V. cholerae* O1 El Tor Inaba (strain T-19479), or *V. cholerae* O139 (strain 4260B) [19]. Heat-inactivated serum was diluted 5-fold, and serial 2 fold dilutions were assayed, with the vibriocidal titer defined as the reciprocal of the highest serum dilution resulting in greater than 50% reduction of the O.D. 600 when compared to control wells without serum. Positive and negative control sera from infected and non-infected individuals were used to ensure consistency across plates. The concentrations of complement and bacteria have been separately optimized for determining the vibriocidal antibody responses to *V. cholerae* O1 and *V. cholerae* O139 [19].

Serum and fecal antibodies specific to CTB, LPS, and TcpA were measured by kinetic ELISAs using methods described previously [10],[20]. 96-well microtiter plates were coated with either purified LPS (250 ng/well), sequentially with GM1 ganglioside (100 ng/well) followed by recombinant CTB (50 ng/well) (gifts of A.M. Svennerholm), or with recombinant TcpA (150 ng/well) [10]. Serogroup/type-specific LPS was derived from the same strains used in the vibriocidal assay by hot-phenol extraction, followed by proteinase, DNAse and RNAse treatment. Plates were incubated with diluted patient sera (1∶50 for LPS ELISA, 1∶100 for TCP, and 1∶200 for CTB), washed, and horseradish peroxidase-conjugated secondary antibodies to either human IgG or IgA were applied (Jackson Laboratories, Bar Harbor, Maine). Plates were developed using 0.1% ortho-phenylene diamine (Sigma, St. Louis, Missouri) in 0.1 M sodium citrate buffer with 0.1% hydrogen peroxide, and optical densities (OD) were read kinetically at 450 nm for 5 minutes at 19-s intervals. ELISA data were normalized across plates using control serum derived from previously infected patients. Antigen specific-IgA in fecal extracts were expressed as a fraction of total IgA in fecal extracts, which was determined by ELISA using an IgA standard (1 mg/ml) derived from human colostrum, as described previously [20].

Serum retinol levels were assayed by high-performance liquid chromatography, and serum zinc levels were assayed by atomic absorption spectrophotometer [16].

### Definition of outcomes in household contacts

Among household contacts, definite *V. cholerae* infection was defined as a positive rectal swab culture for *V. cholerae* during the 21 days of follow-up. Possible infection was defined as a four-fold or greater change in the serum vibriocidal antibody titer and/or the development of diarrheal symptoms in the absence of detection of *V. cholerae* in serial rectal swab cultures. The absence of infection was defined as no positive culture for *V. cholerae*, no significant changes in vibriocidal antibody titer, and no symptoms of diarrhea. Symptomatic *V. cholerae* infection was defined as a positive culture that occurred in association with diarrheal symptoms (3 or more loose stools per day) within 72 hours of developing the positive culture, and asymptomatic infection was defined as a positive culture in the absence of diarrheal symptoms.

### Statistical analyses

Analyses were performed using Stata version 9.0 (Stata Corporation, Inc., College Station, Texas), and SAS version 9.1 (SAS Institute Inc, Cary, North Carolina). Characteristics of the definitely infected household contacts were compared with characteristics of the contacts with no evidence of infection with logistic regression using generalized estimating equations, with an exchangeable correlation matrix, and the reported odds ratios and p values were adjusted for clustering based on household [21]. A multivariate analysis of baseline immunologic characteristics, including age, vibriocidal antibody, and serum anti-CTB, LPS, and TCP IgG and IgA antibodies, was performed with a logistic regression model clustered by household using generalized estimating equations, with the final model determined based on forward selection with a predetermined cutoff criteria of p≤0.05 for inclusion in the model. Odds ratios (OR) are reported in the text and tables with 95% confidence intervals (CI), and all reported p values are two-tailed.

## Results

### Description of the cohort of household contacts

A total of 1077 household contacts of 396 index patients with cholera were enrolled in the study between January 2001 and May 2006. 944 contacts completed 21 days of observation. Of the household contacts that completed follow-up, there were 782 contacts of 304 index patients with cholera due to *V. cholerae* O1, and 162 contacts of 57 index patients with *V. cholerae* O139. Outcomes were defined by whether the household contacts developed diarrhea, had a four-fold change in vibriocidal antibody titer, or had a positive rectal swab for *V. cholerae* and are shown in Table 1. Of the 944 contacts that completed the observation period, 202 developed definite *V. cholerae* infection, defined as a positive rectal swab culture (21%), and of those, 127 (62%) developed diarrhea within 72 hours of their positive culture.

**Table 1:** Outcomes in 944 Household Contacts Completing 21 Days of Follow-up

Category		Number of Contacts	Positive rectal swab culture	4-fold increase in vibriocidal	Symptomatic (diarrhea)
Definite Infection^a^ N = 202	Symptomatic	88	Yes	Yes	Yes
		39	Yes	No	Yes
	Asymptomatic	38	Yes	Yes	No
		37	Yes	No	No
Possible Infection N = 320		39	No	Yes	Yes
		48	No	Yes	No
		233	No	No	Yes
No evidence of Infection^a^ N = 422		422	No	No	No

a Our primary analysis compared household contacts with definite infection, defined a positive rectal swab culture during the 21 days of follow-up, with those with no evidence of infection.

As described above, an additional subset of 229 of the 944 household contacts that completed the 21 days of observation were excluded from the analysis of baseline immunologic characteristics; this included 206 contacts who had diarrhea the week prior to enrollment or a positive rectal swab upon enrollment, and 23 contacts who developed infection with a *V. cholerae* serotype/group that did not match the index case. None of the household contacts required hospitalization or intravenous hydration, although symptomatic individuals were treated promptly with antibiotics and home oral rehydration therapy as needed.

### Age, gender and probability of infection with *V. cholerae*

**Figure 1:**
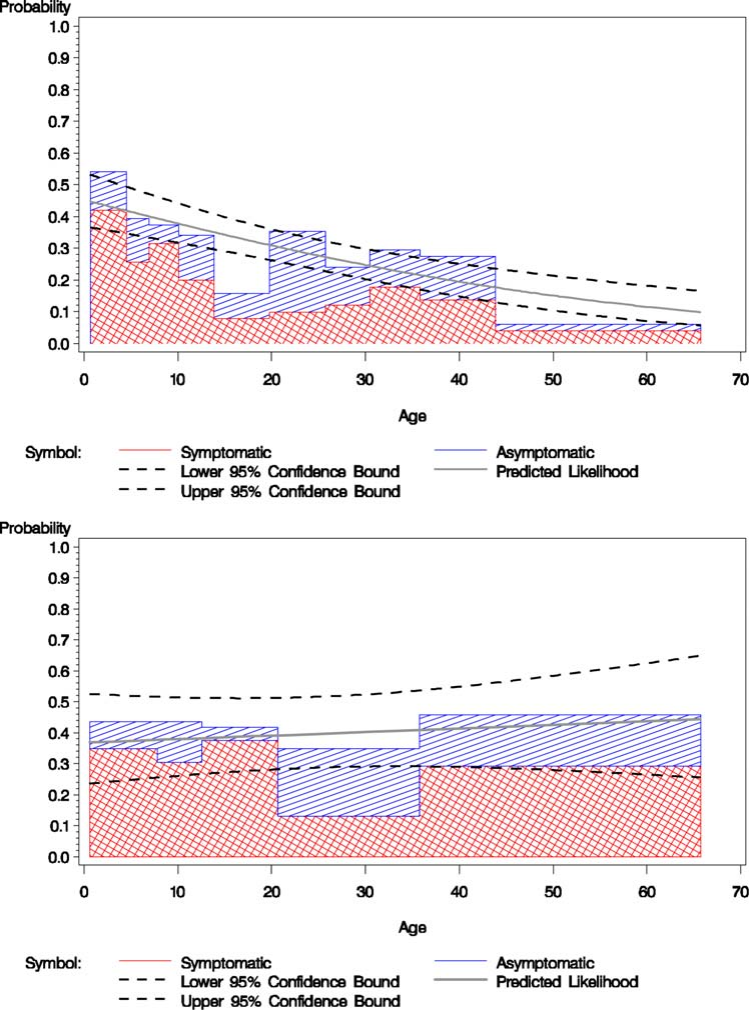
The total proportion of household contacts with definite *V. cholerae* infection by age group. The red-hatched area indicates those with symptomatic infection, the blue-hatched area indicates those with asymptomatic infection. The associated curves represent the predicted likelihood of developing infection by age based on logistic regression modeling. Figure 1A shows the likelihood of *V. cholerae* O1 infection by age bound in deciles (P for predicted model <0.0001). 1B shows the likelihood of developing *V. cholerae* O139 infection according to age bound in quintiles (P for predicted model = 0.6).

Among household contacts of index cases infected with *V. cholerae* O1, age was inversely related to the probability of developing infection (p<0.001; Figure 1). In contrast, no significant association between age and the probability of infection with *V. cholerae* O139 was found (p = 0.6).

The household contacts with the highest risk of *V. cholerae* O1 infection were young children. In children 5 years of age or younger, the odds of developing infection with *V. cholerae* O1 were 2.7 times that of older individuals (P<0.001, 95% CI 1.61–4.49). Children 5 years or younger also had a higher likelihood of developing symptomatic illness if infected with either serogroup (OR = 2.7, P = 0.03, 95% CI 1.10–7.43). No association between gender and susceptibility to infection with *V. cholerae* of either serogroup was found.

### Immunologic markers and probability of infection with *V. cholerae*

We assessed the association between a number of baseline immunologic markers and risk of *V. cholerae* infection in this cohort of household contacts. Associations between serologic markers and the risk of infection are listed in Table 2. As described previously, baseline vibriocidal antibody titer predicted protection from subsequent infection with *V. cholerae* O1 (OR 0.82 for each doubling of titer, 95% CI 0.74–0.92) but not from *V. cholerae* O139 (OR 0.97, 95% CI 0.82–1.15) [7]. However, the baseline vibriocidal titer did not predict the risk of developing symptomatic illness among those who were infected with *V. cholerae* O1 (OR 0.90, 95% CI 0.75–1.07).

**Table 2:** Immunologic correlates of protection from infection with *V. cholerae* in household contacts

Immunologic Marker	Serogroup	Mean Baseline Antibody Levels (±SE) a		Crude Odds of *V. cholerae* infection per doubling^b^	P	95% CI	
		Infected	Not-infected				
		N = 94	N = 383				
**Vibriocidal**	All	5.1 (±0.31)	6.6 (±0.15)	0.84	<0.001	0.77	0.93
	O1	5.7 (±0.36)	7.1 (±0.14)	0.82	0.001	0.74	0.92
	O139	3.6 (±0.42)	3.8 (±0.32)	0.97	0.7 (NS)	0.82	1.15
**CTB-IgA**	All	3.5 (±0.14)	4.1 (±0.07)	0.74	0.002	0.60	0.90
	O1	3.5 (±0.16)	4.1 (±0.08)	0.74	0.003	0.60	0.90
	O139	3.4 (±0.33)	4.2 (±0.19)	0.74	0.15 (NS)	0.49	1.12
**LPS-IgA**	All	3.8 (±0.15)	4.2 (±0.06)	0.76	0.009	0.62	0.93
	O1	3.6 (±0.19)	4.2 (±0.07)	0.70	0.007	0.54	0.91
	O139	4.3 (±0.16)	4.4 (±0.15)	0.91	0.6 (NS)	0.62	1.34
**TcpA-IgA**	All	3.6 (±0.10)	4.0 (±0.06)	0.65	0.002	0.49	0.85
	O1	3.5 (±0.15)	3.9 (±0.06)	0.65	0.015	0.46	0.92
	O139	3.8 (±0.10)	4.2 (±0.10)	0.58	0.038	0.34	0.97
**CTB-IgG**	All	6.2 (±0.11)	6.4 (±0.06)	0.84	0.24 (NS)	0.64	1.12
	O1	6.5 (±0.11)	6.6 (±0.06)	0.93	0.56 (NS)	0.73	1.18
	O139	6.1 (±0.29)	6.6 (±0.13)	0.77	0.20 (NS)	0.37	1.23
**LPS-IgG**	All	6.1 (±0.10 )	6.2 (±0.05)	0.88	0.30 (NS)	0.68	1.12
	O1	6.1 (±0.13)	6.2 (±0.05)	0.88	0.35 (NS)	0.66	1.16
	O139	6.2 (±0.19)	6.3 (±0.11)	0.83	0.52 (NS)	0.46	1.48
**TcpA-IgG**	All	5.3 (±0.08)	5.4 (±0.04)	1.07	0.65 (NS)	0.80	1.40
	O1	5.3 (±0.10)	5.4 (±0.04)	1.05	0.75 (NS)	0.76	1.47
	O139	5.2 (±0.10)	5.4 (±0.08)	0.74	0.48 (NS)	0.31	1.72

a Baseline measurements are shown as log_2_ transformed antibody titer (vibriocidal) or relative kinetic ELISA values. The standard error of each mean is noted in parentheses.

b Adjusted only for clustering by household.

A novel finding in our study was that *V. cholerae* antigen-specific serum IgA levels predicted an individual's susceptibility to *V. cholerae* infection. As with the vibriocidal antibody, higher serum levels of LPS-specific IgA were associated with protection from infection with *V. cholerae* O1, but not with *V. cholerae* O139. CTB-specific serum IgA levels were associated with protection from infection with both the O1 and O139 serogroups (the ORs were equivalent), but this finding only reached statistical significance for *V. cholerae* O1 infection. TcpA-specific IgA levels were associated with significant protection against both O1 and O139 serogroups. In contrast with serum IgA results, there were no associations between serum LPS, CTB, or TcpA-specific IgG and outcomes in household contacts.

In a smaller subset of our cohort of household contacts, we also evaluated CTB and LPS- specific IgA levels in fecal extracts. This analysis included 282 contacts that completed 21 days of follow-up prior to June 2002. Fecal levels of antigen specific IgA were not significantly correlated with serum IgA levels to the same antigen. Levels of fecal CTB IgA were not significantly associated with the risk of infection with *V. cholerae*, or with the risk of diarrhea in infected individuals. However, higher levels of LPS-specific antibodies in feces at baseline were significantly associated with a lower likelihood of developing symptomatic disease in individuals infected with *V. cholerae* O1 (OR 0.58 per two-fold increase, p = 0.027, 95% CI 0.36–0.94).

To explore potential confounding between immunologic markers that were associated with protection from infection with *V. cholerae* O1 in the univariate analysis, we performed a stepwise, multivariate logistic regression analysis using generalized estimating equations. In the final model derived, three variables – age, baseline vibriocidal titer, and baseline serum CTB-IgA titer – were significant independent predictors of susceptibility to infection with *V. cholerae* O1 (Table 3). Serum anti-TcpA and anti-LPS IgA antibodies were more highly correlated with the vibriocidal antibody (Spearman's rank correlation coefficient, ρ = 0.20, P = 0.004, and ρ = 0.35, P<0.0001, respectively for TcpA and LPS) and than were anti-CTB-IgA antibodies (ρ = 0.06, P = 0.23), and anti-TcpA and anti-LPS IgA antibody levels were not predictive of susceptibility to *V. cholerae* O1 independently of the vibriocidal antibody titer.

**Table 3:** A multivariate logistic regression model of predictors of protection from infection with *V. cholerae* O1 El Tor in household contacts

Variable	Adjusted OR	95% CI		P
Age	0.98^a^	0.97	1.00	0.02
Vibriocidal	0.86^b^	0.77	0.96	0.007
CTB-IgA	0.79^b^	0.66	0.95	0.01

a Per year of increasing age.

b Per each doubling of antibody titer.

Because baseline TCP-IgA was the only significant predictor of susceptibility to *V. cholerae* O139 and there were a smaller number of observations, a separate multivariate assessment was not feasible for the O139 serogroup.

### Nutritional markers and probability of infection with *V. cholerae*

We evaluated the association between anthropometric markers of nutritional status and susceptibility to infection with *V. cholerae* in children younger than 5 years of age. Height- and weight-for-age were not significantly associated with likelihood of *V. cholerae* infection or symptomatic disease in young children exposed in the household (Table 4).

**Table 4:** Other non-immunologic predictors of susceptibility to infection with *V. cholerae* O1 El Tor in household contacts

Characteristic	Crude Odds Ratio of *V. cholerae* O1 infection	P	95% CI	
Significant predictors				
Retinol Deficient	2.36	0.05	1.00	5.53
Blood Group O	0.54	0.005	0.35	0.83
First Degree Relative of Case^a^	2.90	0.03	1.12	7.52
Non-predictive				
Zinc Deficient	1.02	0.97	0.28	3.7
Height for Age (Z)	0.88	0.30	0.68	1.12
Weight for Age (Z)	0.78	0.30	0.50	1.24
Weight for Height (Z)	0.88	0.66	0.49	1.57

a Compared to household contacts that were not first degree relatives.

To explore the relationship between micronutrient levels and susceptibility to cholera, we also evaluated baseline zinc and retinol levels in a subset of 278 household contacts (including a total of 55 retinol and 129 zinc deficient individuals). Our evaluation was restricted to contacts of patients with *V. cholerae* O1. Zinc deficiency (defined at serum zinc ≤70 µg/dL [22]) was not significantly associated with likelihood of infection with *V. cholerae* O1 or with development of diarrhea. Retinol deficiency (defined as serum retinol ≤20 µg/dL [22]) was associated with a higher risk of infection with *V. cholerae* O1 (Table 4). Furthermore, retinol deficiency was associated with a higher likelihood of developing symptomatic disease if infected: all 9 of the retinol deficient individuals infected with *V. cholerae* O1 developed symptomatic disease, while only 14 of the 27 retinol replete infected individuals developed symptomatic infection (P = 0.05).

### Genetic factors

Consistent with what was previously described in a earlier subset of the current cohort [23], we found that individuals with blood group O were less likely to become infected with *V. cholerae* O1 than non-blood group O individuals (OR 0.54, 95% CI 0.35–0.83, Table 4), but if infected, had greater than twice the odds of developing symptomatic infection (OR 2.13, P = 0.035, 95% CI 1.05–4.33). There was no significant difference in the susceptibility of individuals with blood group O to infection with *V. cholerae* O139, although individuals with blood group O were more likely to develop symptomatic disease if infected with either serogroup.

Because we hypothesized that additional host genetic factors might contribute to susceptibility to cholera, we collected pedigree data on a subset of the households enrolled in the study (beginning in 2003). A total of 259 household contacts that completed 21 days of follow-up were classified based on relatedness to the index case. The analysis included 197 first-degree relatives (i.e. siblings, parents or children) of the index case, and 62 non-first degree relatives. Among this population, individuals who were first-degree relatives of the index case had significantly greater odds of being infected with *V. cholerae* compared to non-related or less closely related contacts in the same household (OR [crude] 2.90, P = 0.03, 95% CI 1.12–7.52, Table 4). This finding was independent of blood group phenotype and age in a multivariate analysis (OR [adjusted] 2.91, P = 0.03, 95% CI 1.11–7.61).

### Microbiologic and environmental factors

Household contacts of index patients infected with *V. cholerae* O139 were more likely to become infected than contacts of patients with *V. cholerae* O1 (OR 1.67, p = 0.015, 95% CI 1.10–2.52). There was no difference in the likelihood of infection between contacts of index patients infected with Inaba (N = 466) versus the Ogawa (N = 366) serotypes of *V. cholerae* O1 (OR 0.95, p = 0.86, 95%% CI 0.60–1.53).

In addition to intrinsic host and microbiologic characteristics, other environmental factors might influence the outcome of exposure to *V. cholerae* within a household. We found that attack rates were markedly higher in individuals living in households that included more than one infected individual (OR 5.50, P<0.001, 95% CI 3.18–9.51). This finding was independent of baseline vibriocidal antibody titers of household contacts, suggesting that variability in risk of infection in households may be due to a common environmental risk and/or common genetic factors influencing household susceptibility.

## Discussion

An improved understanding of factors that influence host susceptibility to cholera may aid in the development and implementation of an effective vaccination program. In this study, we identified novel immunologic markers that predict protection from *V. cholerae* infection in a population in Bangladesh, as well as other host characteristics that modify susceptibility.

The vibriocidal antibody is the only previously described marker of immunity to cholera and is routinely utilized in pilot studies of vaccine efficacy. Here, we show that levels of serum IgA specific to three V. *cholerae* antigens – CTB, LPS and TcpA – also predict protection among household contacts of patients infected with *V. cholerae* O1 El Tor, the current predominant cause of cholera. Interestingly, levels of serum IgG directed against these same antigens did not predict protection, possibly because serum IgA levels better reflect protective immune responses at the intestinal mucosal surface where secretory IgA (sIgA) is the predominant immunoglobulin. We also examined baseline fecal levels of *V. cholerae* antigen specific IgA, although there was no significant correlation of these with baseline serum IgA antibodies to the same antigen. Antigen-specific fecal IgA levels did not correlate with protection from cholera, except for a mild effect of fecal IgA specific to LPS. This might reflect proteolysis of IgA in fecal samples, such that baseline fecal IgA measurement may not adequately represent an accurate level of the response to *V. cholerae* antigens at the mucosal level.

Among the antigens evaluated, responses to TcpA appear to be unique in that they are associated with protection from both the O1 and O139 serogroups. Expression of *tcpA* mRNA is highly up-regulated in *V. cholerae* during human infection [24], yet it remains uncertain to what extent this antigen is present in current killed and live-oral cholera vaccines. A systematic comparison of TcpA responses in naturally-infected individuals compared to vaccine recipients would be important to address this question. Our data suggest that inclusion of TcpA as a vaccine component may be useful in boosting protective immunity across both serogroups.

Studies in the rabbit ileal loop model of cholera suggest that anti-toxin and anti-LPS immune responses are synergistic in the prevention of fluid accumulation [25]. In concordance with this observation, our multivariate analysis showed that both anti-toxin (anti-CTB) and anti-bacterial (vibriocidal antibody) responses were independent predictors of susceptibility to cholera. This finding is also consistent with the results of clinical trials of the whole cell-B subunit vaccine, which suggest a role for anti-CTB responses in conferring additional short term protection compared with whole cell vaccine alone [9]. Our multivariate model also indicated that increasing age predicted immunity from cholera, independent of both anti-CTB IgA and vibriocidal antibody levels, suggesting that additional components of protective immunity remain unidentified.

In addition to adaptive immune responses, we identified other host and environmental characteristics that affected the risk of developing cholera in our study population. Although an association between blood group phenotype and severity of cholera has previously been recognized, we observed an additional familial segregation of susceptibility to cholera within households. In particular, first-degree relatives of cholera patients were significantly more likely to develop infection with *V. cholerae* than less closely related members living in the same household, and this was independent of blood group. Such increased susceptibility might be related to closer contact or shared behaviors between first degree relatives, or could reflect additional genetic components of susceptibility to cholera.

We found direct evidence to support the importance of nutrition in susceptibility to infection with *V. cholerae*. Increasing levels of retinol, but not zinc, were associated with decreased susceptibility to both infection and symptomatic disease. This observation underscores the importance of retinol supplementation in young children in developing countries where cholera is endemic.

Our study has a number of limitations. Although we demonstrated that cholera antigen-specific serum IgA levels correlate with protection from infection with *V. cholerae*, it is possible that these immunologic markers, like the vibriocidal antibody, are surrogates for protective immune responses localized at the mucosal surface. Thus, the inability to measure intestinal levels of sIgA using non-invasive techniques remains a major limitation and necessitates the discovery of better proxy measurements of mucosal immunity. It is also possible that a portion of household contacts in our study were infected prior to onset of symptoms of the recognized household index case. We accounted for this by excluding from assessment of baseline immunologic characteristics those contacts reporting diarrhea in the week prior to enrollment or having a positive rectal swab upon entry into the study.

Taken together, our data suggest that immunologic, genetic, and nutritional characteristics of individuals all contribute to human susceptibility to infection with *V. cholerae* in a household. Although it is hypothesized that immune responses at the mucosal surface are the primary mediators of protection from *V. cholerae*, the specific antigens to which these responses are directed have not been identified. Our observation that levels of serum IgA (but not serum IgG) directed at certain *V. cholerae* antigens are associated with protection from infection underscores the need to better understand anti-*V. cholerae* immunity at the mucosal surface. Such understanding would be critical in designing and evaluating improved vaccines against *V. cholerae*.
